# Integrating wastewater and randomised prevalence survey data for national COVID surveillance

**DOI:** 10.1038/s41598-024-55752-9

**Published:** 2024-03-01

**Authors:** Guangquan Li, Peter Diggle, Marta Blangiardo

**Affiliations:** 1 Applied Statistics Research Group, Department of Mathematics, Physics and Electrical Engineering, Northumbria University, Newcastle upon Tyne, NE1 8ST UK; 2 Lancaster University, Lancaster, LA1 4YW UK; 3 MRC Centre for Environment and Health, Imperial College London, St Mary’s Campus, Norfolk Place, London, W2 1PG UK; 4 Turing-RSS Health Data Lab, London, UK

**Keywords:** Epidemiology, Epidemiology, Epidemiology, Epidemiology

## Abstract

During the COVID-19 pandemic, studies in a number of countries have shown how wastewater can be used as an efficient surveillance tool to detect outbreaks at much lower cost than traditional prevalence surveys. In this study, we consider the utilisation of wastewater data in the post-pandemic setting, in which collection of health data via national randomised prevalence surveys will likely be run at a reduced scale; hence an affordable ongoing surveillance system will need to combine sparse prevalence data with non-traditional disease metrics such as wastewater measurements in order to estimate disease progression in a cost-effective manner. Here, we use data collected during the pandemic to model the dynamic relationship between spatially granular wastewater viral load and disease prevalence. We then use this relationship to nowcast local disease prevalence under the scenario that (i) spatially granular wastewater data continue to be collected; (ii) direct measurements of prevalence are only available at a coarser spatial resolution, for example at national or regional scale. The results from our cross-validation study demonstrate the added value of wastewater data in improving nowcast accuracy and reducing nowcast uncertainty. Our results also highlight the importance of incorporating prevalence data at a coarser spatial scale when nowcasting prevalence at fine spatial resolution, calling for the need to maintain some form of reduced-scale national prevalence surveys in non-epidemic periods. The model framework is disease-agnostic and could therefore be adapted to different diseases and incorporated into a multiplex surveillance system for early detection of emerging local outbreaks.

## Introduction

During the COVID-19 pandemic, several countries ran large-scale, randomised prevalence studies to monitor the spatio-temporal evolution of the epidemic. In the UK, these include the REal-time Assessment of Community Transmission (REACT) study^1^ and the Office for National Statistics (ONS) COVID-19 Infection Survey (CIS)^2^, both of which have since been discontinued. However, continued monitoring is needed in order to understand the continuing circulation of this and other viruses and as a component of an ongoing public health surveillance system for early identification of any future local outbreaks.

Randomised prevalence studies represent a gold standard for health surveillance but are expensive. A likely future scenario is that an affordable surveillance system will need to combine relatively sparse prevalence data with more abundant, lower-cost, non-traditional disease metrics. Wastewater-based epidemiology (WBE) provides promising candidates for this role. Compared to other diagnostic testing instruments, wastewater sampling is non-invasive and is cheaper to conduct. The population-based nature of wastewater sampling alleviates the issue of selection bias that is often encountered in conventional diagnostic testing^3,4^, as all residents within the catchment area of a sewage treatment work contributing towards the data collection process. Moreover, in the case of COVID, wastewater sampling captures data from both symptomatic and asymptomatic cases. WBE has been demonstrated to be a cost-effective tool for monitoring disease trends during the COVID-19 pandemic, both at city or regional level^5–11^ and as a nationwide approach in a number of countries^12–14^.

The wastewater data used in this study come from Li et al^15^., who used data on SARS-CoV-2 viral load in wastewater across a network of 303 sewage treatment sites in England to obtain probabilistic predictions at high spatial-temporal resolution covering the whole country (see the Methods section). Their study also included an exploratory analysis of the relationship between wastewater viral load and disease prevalence and found that these two metrics showed good qualitative agreement, but that the quantitative relationship between them appeared to vary both in space and in time^15^. This implies that wastewater alone cannot be used to estimate prevalence, which was also observed in a recent study^16^.

In this paper we develop a statistical framework for disease surveillance that combines wastewater viral load and disease prevalence data under the following scenario:

(i) wastewater data continue to be collected through the England-wide sewage treatment works network and are used to obtain predictive distributions of the SARS-CoV-2 viral load at Lower Tier Local Authority (LTLA) level using the wastewater model described in^15^;
(ii) REACT survey data are used to obtain predictive distributions of de-biased COVID-19 prevalence at LTLA level using the methodology described in^17^, but *only up to a certain point in time*;
(iii) prevalence estimates at national level continue to be available throughout the period of study.

Our aim is to formulate a data integration model for wastewater viral load and disease prevalence estimates at both fine-scale (here, LTLA) and coarse-scale (here, national) spatial resolutions to enable fine-scale prediction of disease prevalence to continue when only coarse-scale prevalence data are available.

**Figure 1:**
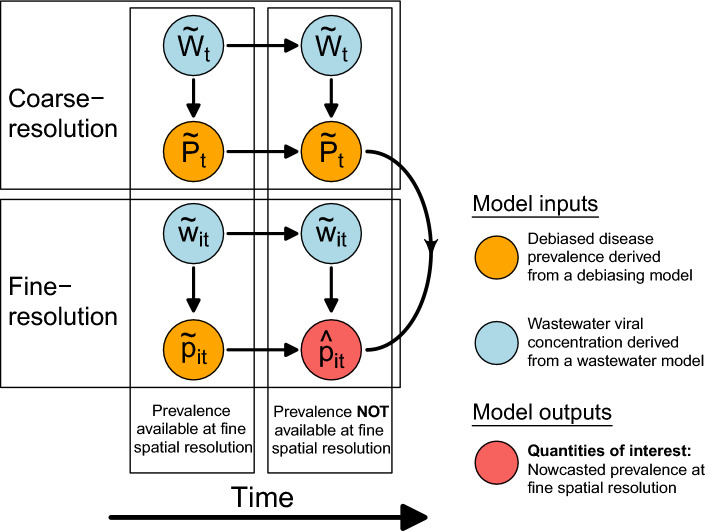
Schematic representation of the data integration model for wastewater viral load and disease prevalence at LTLA-level and National-level spatial resolutions. Each circular node represents a random variable associated with a probability distribution. The $\sim$ overscript indicates that the associated node is a probabilistic output from either the debiasing model or the wastewater model. The quantities of interest from our data integration approach are ${\hat{p}}_{it}$, the nowcasted disease prevalence at a fine spatiotemporal scale. See Methods Section for detail.

We formulate our model by treating COVID-19 prevalence as a spatiotemporal outcome with viral load in wastewater and other observable covariates (e.g. small-area sociodemographic characteristics) as predictors. In order to capture the dynamic relationship between prevalence and wastewater metrics, we formulate the regression coefficients in the model as spatially and temporally varying random effects, and fit the model using Bayesian inference with weakly informative priors for the model parameters. Figure 1 is a schematic representation of the overall model structure.

## Results

### Space-time association between COVID prevalence and viral concentration in wastewater

**Figure 2:**
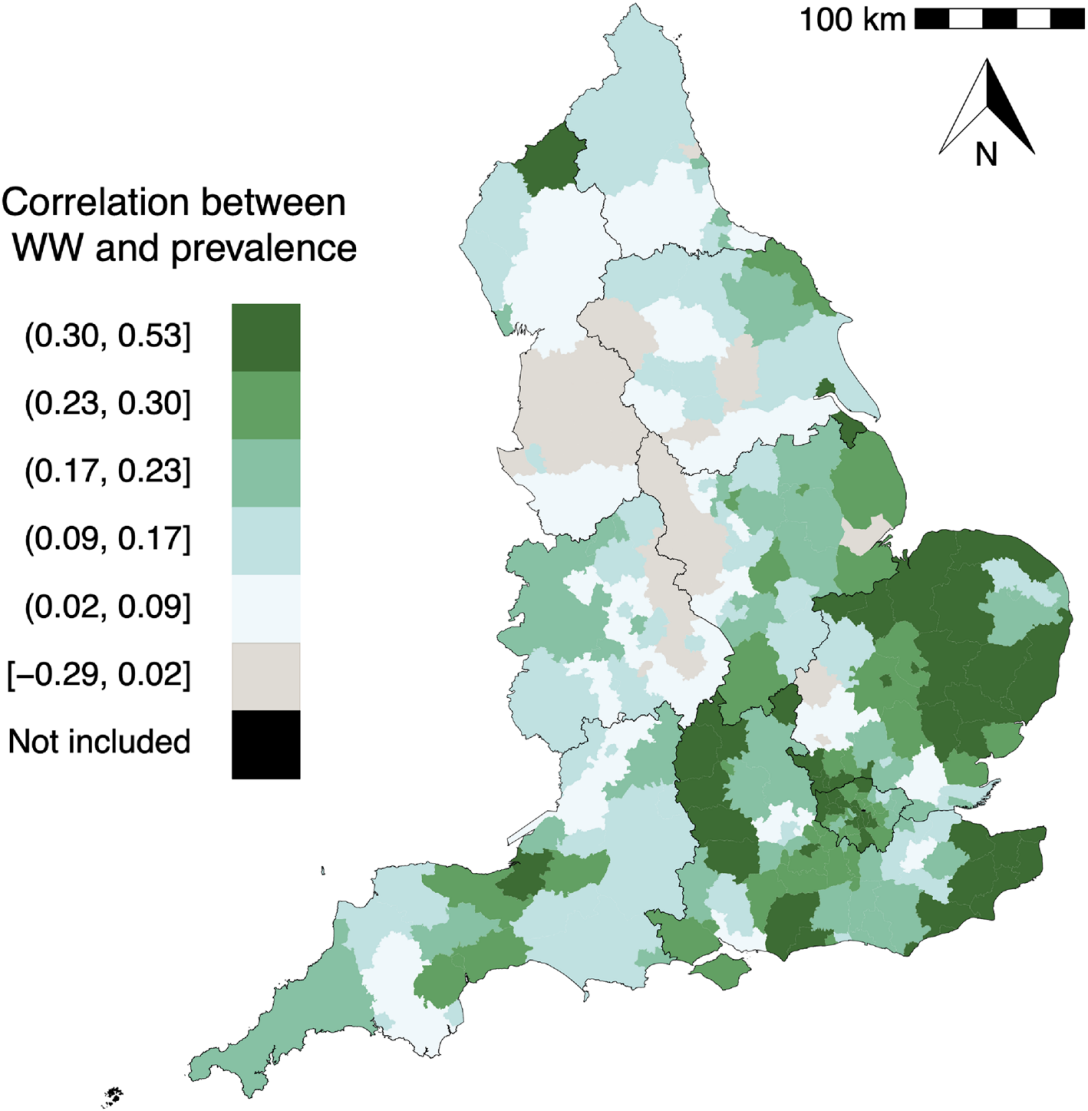
The spatial distribution of the correlation (Kendall’s tau) between wastewater viral load and de-biased COVID prevalence at LTLA level over the study period. Due to the absence of the de-biased prevalence, this study excludes the two LTLAs in black, City of London and Isle of Scilly, a group of small islands in the southwestern tip of England. Roughly 50 LTLAs fall into each colour band, except the one in black. The digital vector boundaries for Local Authority Districts and for Regions in England were obtained from the Office for National Statistics Open Geography Portal^18^ (Source: Office for National Statistics licensed under the Open Government Licence v.3.0 and contains OS data $\copyright$ Crown copyright and database right 2022). The map in this figure was produced in R^19^ (version 4.3.1). The R script to produce this figure can be found on the GitHub repository https://github.com/gqlNU/wwprev. .

Over the 307 LTLAs that cover the whole of England, 260 show a positive correlation between estimates of wastewater viral concentration^15^ and de-biased COVID prevalence^17^ over the study period from June 1, 2021 to March 30, 2022, indicating the potential for using wastewater data as a predictor of disease prevalence. However, the strength of the correlation varies substantially over space. LTLAs in East Midlands and East of England generally show a stronger positive correlation than those in North West and parts of the North East and West Midlands regions (Fig. 2). Note that this study includes 307 out of the total 309 LTLAs in England as the de-biased prevalence estimates are unavailable from Nicholson et al. 2022^17^ for two LTLAs, City of London and Isle of Scilly.

### Nowcasting weekly LTLA-level prevalence

To evaluate predictive performance, we fitted two models, (i) where the relationship between wastewater viral load and prevalence varies in space and time (full model) and (ii) where the relationship varies only in space (simplified model). We used all the prevalence and the wastewater data over the period of 40 weeks to conduct a cross-validation study. Specifically, the LTLA- and the national-level prevalence data and the LTLA- and the national-level wastewater data over the first 20 weeks are made available to the model to learn about the space-time variation in prevalence and the spatially- and temporally-varying wastewater-prevalence relationship. Over the subsequent 20 weeks, wastewater data (LTLA-weekly and national-weekly) continue to be available, as well as the national-weekly prevalence but the LTLA-weekly prevalence are hidden from the model. The aim of the modelling is to nowcast the weekly prevalence at the LTLA level over the latter 20 weeks (see the Methods sections for more details). To formally compare the predictive accuracy over the validation period, we calculated the root mean square error (RMSE) and the average coverage of nominal 95% credible intervals. Both models give the same RMSE of 0.271. The 95% coverage rates are 96.0% from the full model and 94.4% from the simplified model, indicating both models deliver reliable estimates of predictive uncertainty. However, comparing the width of the intervals (Figure 2 in Supplementary) we see that the majority of LTLAs have narrower intervals under the simplified model, suggesting more precise estimates. Thus, from now on we will only report the results of this model and refer to it as the *data integration model*.

#### Removing national prevalence

**Figure 3:**
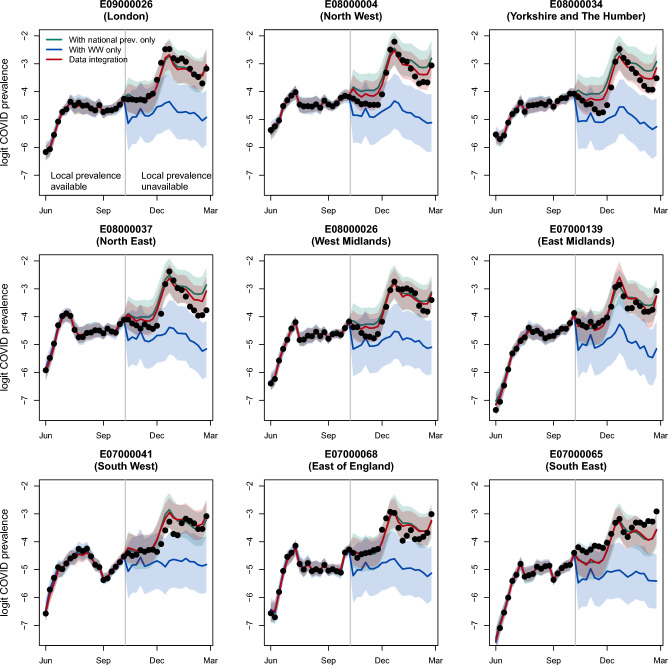
Comparison of the logit-transformed COVID prevalence. The black dots represent the observed prevalence; the dark green line is the nowcast prevalence using the national prevalence estimates only (posterior mean + 95% uncertainty band); the blue line is the nowcast prevalence using wastewater data only; the red line is the nowcast prevalence obtained through the integration of wastewater data and national level prevalence. We show a randomly selected LTLA in each of the 9 English regions.

The importance of incorporating the national prevalence in the data integration model is evident in Fig. 3, where for a random selection of LTLAs we compare the observed disease prevalence (dots) against the predicted prevalence obtained using only the wastewater data (blue line, model described in Supplementary Section A) and using the wastewater data together with national-level prevalence estimates (red line, model described in Eqs. 1 and 2 in the Methods Section). Across LTLAs the model without the national prevalence tends to consistently underestimate prevalence, while incorporating this information in the model leads to substantial improvement with a majority of the observed (true) prevalence values lying within the estimated 95% intervals.

#### Removing wastewater estimates

To demonstrate the added value of wastewater data we also compared the LTLA-level predictions from the data integration model against those from a model using only the national prevalence estimates. Table 1 summarises the results as regional average values of the RMSE and the width of the 95% credible intervals. The data integration model gives a smaller RMSE for all regions of England except East of England, where there is very little difference, and narrower 95% credible intervals. The addition of wastewater data not only improves the accuracy of the prevalence estimates but also reduces uncertainty. The value of incorporating wastewater in the prevalence nowcast is also evident in Figure 3, most noticeably for the illustrative LTLAs in the North West, Yorkshire and The Humber and the North East.

**Table 1:** Region-level predictive performance using national-level prevalence estimates alone (Model A) and in conjunction with LTLA-level wastewater viral load estimates (Model B).

Region	Model A		Model B	
	RMSE	WIDTH	RMSE	WIDTH
East Midlands	0.265	1.215	0.237	1.049
East of England	0.224	1.215	0.229	1.043
London	0.273	1.215	0.264	1.008
North East	0.444	1.212	0.340	1.027
North West	0.373	1.209	0.314	1.031
South East	0.321	1.214	0.287	1.030
South West	0.310	1.219	0.294	1.038
West Midlands	0.268	1.213	0.230	1.028
Yorkshire and the Humber	0.364	1.211	0.262	1.047
England-wide	0.308	1.214	0.271	1.034

Performance measures are root mean square error (RMSE) and width of pointwise 95% credible intervals (WIDTH), averaged over the 20 week validation period and all LTLAs within each region.

### Adding covariates

As the data integration model is flexible, it can readily be extended to incorporate observable covariates that might show a relationship with both disease prevalence and wastewater viral concentration, hence potentially improving the nowcasting. Here, as an illustration, we considered two LTLA-level covariates: the Index of Multiple Deprivation (IMD) and proportion of the Black, Asian and Minority Ethnic (BAME) population, both of which have been found to be associated with prevalence^20^ and were used in estimating local wastewater viral load^15^. Their inclusion gave no discernible improvement in predictive performance with a RMSE remaining at 0.271 and the average width of the 95% credible intervals being 1.027, similar to that from the model without these two covariates (Model B in Table 1).

### Informing the design of a cost-effective data collection strategy for national surveillance

The scenario in which national-level prevalence estimates continue to be available every week is but one of a number of possibilities. We believe that this model framework can be used to evaluate the advantages and drawbacks of several hypothetical surveillance strategies, assuming different spatial and temporal data availability for the coarse level prevalence estimates, aiming to find the most cost-effective one that produces accurate nowcasting. Table 2 summarises the average LTLA-level RMSE for the data integration model, for each English region, under the current scenario and three alternative ones: national-level data available every 5 weeks; regional-level data available every week; regional-level data available every 5 weeks. As expected the smallest RMSEs are estimated for the model with regional weekly prevalence, the scenario with the highest spatio-temporal resolution. Reducing the availability of the data either in space (regional to national) or time (weekly to every 5 weeks) leads to substantial increases in the RMSE that are broadly consistent across all nine regions, with the majority suggesting that reducing the spatial granularity (from regional to national) might be preferable to reducing the temporal one (from weekly to one in 5 weeks). Comparisons of this kind can inform future discussion of the cost-effectiveness of different options for continuing collection of prevalence data.

**Table 2:** Regional average root mean square error of predictions over the 20 week validation period under four scenarios for continuing collection of prevalence data to provide prevalence estimates at a coarse spatial and/or temporal resolution as input to the data integration model.

Region	Regional weekly	National weekly	Regional 1 in 5 weeks	National 1 in 5 weeks
East Midlands	0.187	0.237	0.345	0.374
East of England	0.188	0.229	0.324	0.345
London	0.168	0.264	0.249	0.308
North East	0.141	0.340	0.391	0.538
North West	0.250	0.314	0.395	0.468
South East	0.208	0.287	0.289	0.271
South West	0.198	0.294	0.286	0.388
West Midlands	0.209	0.230	0.330	0.367
Yorkshire and the Humber	0.245	0.262	0.366	0.407

## Discussion

In this paper, we have proposed a statistical framework to downscale disease prevalence estimates from national to local level, exploiting the availability of the spatially granular data on viral load in wastewater that can be collected at much lower cost than spatially granular prevalence survey data. Our approach can capture spatially and temporally dynamic relationships between disease prevalence and wastewater viral load. It accounts for the uncertainty inherent in the estimates of the prevalence and of the wastewater viral load, and can incorporate additional spatially and/or temporally varying covariates where these can be shown to improve the prediction of local disease prevalence.

We have developed and applied our framework in the specific context of COVID prevalence in England. Our results have demonstrated the added value of incorporating fine-scale wastewater data to improve the accuracy and reduce the uncertainty of the local prevalence nowcast. The developed framework is general and could be applied to any disease whose patients shed disease-specific genetic material into their local wastewater network. Similarly, the framework could be used in any geographical setting in which georeferenced data on disease prevalence and wastewater viral load are available. In any new setting, the model would need to be calibrated to quantify the association between disease prevalence and wastewater viral load in the specific geographical setting. When applying the proposed framework to a new setting, the following pipeline could be followed. The wastewater model by^15^ is applied to the wastewater viral load measurements at the sewage treatment work level to obtain estimates, with associated uncertainty, of wastewater viral load at the spatiotemporal scale that aligns to the one available for the disease prevalence (e.g. the LTLA-weekly level in our COVID application). The data integration models developed here are applied to infer the spatiotemporal variation in prevalence and the spatially and temporally varying wastewater-disease relationships when local prevalence data are available. The inferred relationships are then employed to nowcast local prevalence when data on disease prevalence is only available at a coarser spatial level.

In the COVID application, our joint model identifies a relationship between wastewater viral load and prevalence that is variable in space but appears to be quite stable in time over our study period. Nevertheless, the relationship is likely to change in the long run, calling for some level of periodic re-calibration that would require the availability of local-level prevalence data. This points to the importance of maintaining relatively low-intensity randomised survey programmes for viral disease prevalence in non-epidemic periods. Where this is infeasible for reasons of cost, as might apply in low-resource settings, the wastewater component of our joint model could still be used to monitor *changes* in the wastewater signal that could trigger targeted disease interventions in potential hot-spots.

Our nowcasts are obtained by sampling from the predictive distribution of the underlying disease prevalence process. This has two important consequences. Firstly, we can quantify the uncertainty in the results, which allows the user to distinguish between random fluctuations and genuine changes in behaviour. Secondly, we can sample from whatever property of the disease prevalence process that is relevant to public health decision-making; for example we can calculate the probability that local prevalence exceeds a previously agreed intervention threshold.

We might expect model performance in nowcasting local prevalence to improve with the addition of variables which might act as effect modifiers between wastewater viral load and disease prevalence. We included two local population characteristics, % BAME and IMD, but did not find any improvement in nowcast performance. Two possible explanations for this are, firstly, that the spatially varying random effects in the model are able to capture variation that would otherwise be attributed to the covariates, secondly, that their effects are adequately captured by their inclusion in the sub-model for wastewater viral load^15^. This suggests that, although these variables might indeed be associated with disease prevalence, when the aim is prediction a parsimonious model that only includes random effects might be preferable. We emphasise that while we showed that the model can easily handle covariates, selection of covariates is disease specific and needs to be carefully evaluated on a case-by-case basis.

We used RMSE, coverage rate and average interval width as a set of general measures of comparative predictive performance to evaluate different model specifications, but in any specific application we strongly advocate reporting predictive probabilities that relate directly to public health decision-making, such as exceedance of a pre-specified threshold of local prevalence or its rate of change. At the very low levels of prevalence that would be expected outside epidemic periods, it would be extremely difficult for any empirical surveillance system to yield precise estimates of absolute prevalence, but nor are precise estimates needed at such times, only a high predictive probability that prevalence is above a level that would be cause for public health concern. We believe that our understanding of the underlying disease process can be improved by synthesising information from the available data sources from randomised surveys, diagnostic testing and wastewater sampling through the use of the proposed data integration approach.

Our model for the COVID application has some limitations. Firstly, it does not allow for geographical variation in lead-lag relationships that, if present, might explain some of the regional variation in the wastewater-prevalence relationship beyond that accommodated by the LTLA-level variation. Secondly, the true local prevalence is unlikely to be fully described using local wastewater concentrations in conjunction with coarser-level prevalence survey data. Other more finely resolved data, if available, could be included as additional model components. For example, in the UK setting these could include GP presentations with non-specific viral symptoms, non-prescription medication sales, hospitalisation and mortality from national administrative registries or calls to the phone-in triage service NHS111 (https://111.nhs.uk), which includes a record of the full UK post-code (typically resolved to the level of a single street) of each caller. Thirdly, even high-quality randomised surveys, which in principle can deliver unbiased estimation of disease prevalence, are susceptible to non-response bias. In our COVID application, we sought to minimise this by using what we believe to be the best available, de-biased prevalence estimates, from^17^. Nevertheless, this shows the importance of maintaining a consistent sampling protocol over time, so as to enable unbiased estimation of relative changes in prevalence, if not their absolute values. Finally, although our focus is on modelling the wastewater-prevalence relationship at the population level, we acknowledge individual-level variations in the shedding dynamics, e.g. the amount of virus shed and the duration of shedding^21^. In addition, the STW network in England, while covers a majority of the country’s population, does not capture a small percentage of households that are not within any STW catchment (e.g. with onsite sanitation systems). However, this limitation of the wastewater data could be addressed by incorporating other spatially refined data sources as mentioned above, highlighting the value of the proposed data integration approach.

Large-scale randomised surveys are extremely costly and unlikely to be run at scale other than during a pandemic. Our results show that for routine health surveillance, a cost-effective strategy would be to combine reduced-scale prevalence surveys with a cheaper proxy variable that can be measured at a fine spatial resolution. We have investigated one such scenario, in which prevalence surveys are only available at national level and the proxy variable is wastewater viral load. We have also shown that changing the spatial and/or temporal granularity of the surveys has an important effect on the bias in the nowcasted prevalence. Other strategies for reducing costs during non-epidemic periods would be to maintain a spatially granular national prevalence survey but with smaller sample sizes, or to continue with large sample sizes over a sentinel network. How these affect the results would need to be investigated, but is out of the scope of the present paper.

To conclude, wastewater is a promising data source for disease surveillance. We have shown how it can be used to predict COVID prevalence at local level when anchored to disease data at a coarser resolution. The approach is transferable to other diseases and can be extended to include more than two data sources. The data integration approach presented here provides a foundation for building a cost-effective multiplex health surveillance system, to flag where and when additional resources need to be swiftly deployed to reduce the disease burden on the health system and on the population.

## Methods

### Estimating the SARS-CoV-2 viral concentration in wastewater at the LTLA-weekly scale

Estimates of LTLA-weekly SARS-CoV-2 viral concentration in wastewater are available as the output of the modelling framework presented in^15^. In England, weekly measures of viral concentration in wastewater, reported as the (log-transformed) number of SARS-CoV-2N1 gene copies per litre (gc/L) of wastewater, were made available via the Environmental Monitoring for Health Protection (EMHP) wastewater surveillance programme^22^ across the 303 sewage treatment works (STWs) between 1 June 2021 and 30 March 2022. The STW network covers approximately 74% of the population in England^23^. However, a key challenge is that the STW catchment boundary is misaligned with that of the LTLAs, the spatial resolution at which the prevalence estimates are available. In the work^15^, a Bayesian spatio-temporal geostatistical model was developed to estimate weekly wastewater viral concentration at the LTLA level covering the entirety of England. There are two key components in the wastewater model. The first key component in the model evaluates the relationship between the measured weekly viral concentration with a set of covariates including population socio-demographics, land use and genomic information of the virus at the STW level. All the covariates included are available at the Lower Super Output Area level (LSOA, $n=$32844), apart from the genomic ones which are available at weekly national level. The LSOA-to-STW lookup from^24^ was used to map the LSOA covariates to the STW level. The second key component of the wastewater model is the incorporation of a collection of random effect terms, each specified via a Gaussian random field, to capture the space-time variations in the wastewater measurements. These include the overall temporal pattern, the regional and the between-site variations, and the local variation in the viral measurements that changes smoothly over both space and time. Based on the learnt wastewater-covariate relationship and the fitted space-time correlation structures, the model predicts wastewater viral concentration, together with the associated uncertainty, for all of the 32844 LSOAs in England. We then aggregated the LSOA-weekly predictions, weighted by population, to the Lower Tier Local Authority (LTLA) level to match the spatial resolution of the available COVID-19 de-biased prevalence estimates (see below for a summary of how the de-biased prevalence is obtained). Similarly, we also aggregated the LSOA-weekly predictions to obtain the population-weighted weekly viral concentrations at the national level. All wastewater viral concentration estimates are probabilistic, with associated predictive distributions. An important feature of our data integration framework is the incorporation and propagation of such uncertainty, the detail of which shall be discussed in the Implementation Section.

### Estimating the LTLA-level and the national-level COVID-19 prevalence

Estimates of COVID-19 prevalence at the LTLA-weekly level are obtained from^17^. Their methodology combines data from targeted diagnostic testing and from national randomised prevalence surveys to produce de-biased prevalence estimates at a fine spatiotemporal resolution (LTLA-weekly) for the whole of England. The data on diagnostic testing used came from Pillar 1 and Pillar 2 polymerase chain reaction tests conducted in England^25^ and the survey data were from the REACT study^1^. The methodology incorporates these two complementary data sources to address their respective limitations. While data from randomised surveys provide accurate estimates of prevalence, statistical precision is lacking when making inference at a fine spatiotemporal scale. Data from targeted testing, on the other hand, are available at high spatiotemporal resolution as these testing programmes cover the wider population but they suffer from ascertainment bias. That is because targeted testing is directed at people who are at increased risk of being infected (e.g. people with COVID symptoms and frontline workers). The method by^17^ corrects for the ascertainment bias through combining accurate prevalence information from the randomised surveys with the targeted testing data. This bias correction is then applied to the local targeted testing data to obtain the bias-corrected prevalence estimates at the LTLA-weekly level.

The outputs from the de-biased model are a set of posterior predictive distributions for ${\tilde{p}}_{it}$, the logit-transformed LTLA-weekly de-biased prevalence. More specifically, for each prevalence estimate, we have ${\tilde{p}}_{it}\sim N(p_{it},\sigma ^2_{it})$ with the reported posterior mean $p_{it}$ and posterior variance $\sigma ^2_{it}$. When modelling the prevalence in the data integration framework, we need to acknowledge the uncertainty associated with the estimates.

We derived ${\tilde{P}}_t$, the population-weighted national-weekly de-biased logit-transformed prevalence, via

$$\begin{aligned} {\tilde{P}}_t = \text{ logit }\left( \frac{\sum _{i=1}^{307} \text{ pop}_i \cdot \text{ expit }({\tilde{p}}_{it})}{\sum _{i=1}^{307} \text{ pop}_i}\right) , \end{aligned}$$

where $\text{ pop}_i$ is the population of the *i*th LTLA and $\text{ expit }(a) = 1/\{1+\exp (-a)\}$, the inverse of the logit transformation. To carry the uncertainty in ${\tilde{p}}_{it}$ over to ${\tilde{P}}_t$, we computed the above transformation on multiple sets of values for ${\tilde{p}}_{it}$ randomly sampled from their predictive distributions. We denote by $P_t$ and $\sigma ^2_t$ the mean and variance of the resulting distribution for ${\tilde{P}}_t$. Again, in the model specification, we need to account for the uncertainty associated with the national prevalence estimates.

### A modelling framework for data integration

The core feature of our data integration model is that it infers the space-time variation in disease prevalence and the relationship between wastewater viral load and disease prevalence when both metrics are available at the LTLA-weekly level. We then use the inferred space-time variation and the learned wastewater-prevalence relationship to nowcast LTLA-level prevalence using the LTLA-weekly wastewater estimates and the national-weekly prevalence estimates. To integrate the data at two different spatial resolutions, the approach consists of two interconnected sub-models, the national-level and the LTLA-level sub-models, within a Bayesian hierarchical modelling approach that flexibly captures the complex wastewater-prevalence relationship and allows the incorporation and propagation of uncertainty. The formulations of the two sub-models are detailed as follows.

#### The national-level sub-model

We model $P_t$, the logit-transformed de-biased disease prevalence, as

1 $$\begin{aligned} P_{t}\sim & {} \text{ Normal }(\mu _{t},\sigma ^2_{t})\nonumber \\ \mu _{t}= & {} \alpha + B_t + (d+D_t) \cdot {\tilde{W}}_t \end{aligned}$$

In (1), both $P_t$ and $\sigma ^2_{t}$ are the mean and variance of the distribution for the logit-tranformed national-weekly prevalence as defined in the Section on COVID-19 prevalence above and both quantities enter the model as data. The inclusion of $\sigma ^2_{t}$ in the data likelihood accounts for the uncertainty associated with the national prevalence estimates. The underlying disease prevalence, $\mu _t$, is then modelled as a combination of an intercept ($\alpha$), a set of temporal random effects ($B_t$) that capture the weekly variation in disease prevalence and a component ($(d+D_t)\cdot {\tilde{W}}_t$) that quantifies the link between wastewater viral concentration and prevalence. More specifically, we included $D_t$, a set of temporal random effects, to allow the wastewater-prevalence relation to vary over time. The term ${\tilde{W}}_t$ denotes the wastewater viral concentration for week *t* at the national level. We will discuss how it is defined in the Implementation Section in order to account for the uncertainty associated with the wastewater estimates.

To flexibly describe the temporal variation in disease prevalence, we used a first-order random walk prior on $B_t$:

$$\begin{aligned} B_t \mid B_{t-1} \sim \text {Normal}(B_{t-1},\sigma ^2_B). \end{aligned}$$

For $D_t$, we used a second-order random walk,

$$\begin{aligned} D_t \mid D_{t-1}, D_{t-2},\ldots \sim \text {Normal}(2 D_{t-1} - D_{t-2},\sigma ^2_D), \end{aligned}$$

to impose a smooth temporal pattern on the variation in the wastewater-prevalence regression relationship.

We assigned weakly informative priors for all model parameters. Specifically, we used a uniform distribution between 0 and 10 for each of the random effect standard deviations ($\sigma _D$ and $\sigma _B$) and a Normal distribution with mean 0 and variance 10^6^ for $\alpha$, the intercept, and for *d*, the time-invariant wastewater-prevalence coefficient.

#### The LTLA-level sub-model

We model the logit-transformed LTLA-weekly de-biased prevalence as

2 $$\begin{aligned} p_{it}\sim & {} \text {Normal}(\mu _{it},\sigma ^2_{it})\nonumber \\ \mu _{it}= & {} (\alpha + U_i) + (B_t + V_{it}) + (d + D_t + M_i) \cdot {\tilde{w}}_{it}. \end{aligned}$$

Similarly to the national-level sub-model, we included the variance $\sigma _{it}^2$ in the likelihood to acknowledge the uncertainty associated with the prevalence estimate $p_{it}$.

In Eq. 2, the space-time variation of the underlying local disease prevalence, $\mu _{it}$, is decomposed into an overall average, $\alpha$, the between-LTLA variation, $U_i$, the national trend, $B_t$, a term $V_{it}$ that accounts for local departures from the national trend, and terms $(d + D_t + M_i) \cdot {\tilde{w}}_{it}$ for the effects on prevalence of local wastewater viral concentration. The specification of ${\tilde{w}}_{it}$ will be discussed in the Implementation Section. There are two main features in the formulation of the local sub-model. First, we included two sets of random effects, $U_i$ and $M_i$, to learn about the local variation in the disease prevalence and in the wastewater-prevalence relationship, respectively, when local prevalence estimates are available. Second, the local sub-model shares a number of terms with the national sub-model, namely $\alpha$, $B_t$, *d* and $D_t$. In this way, when nowcasting local prevalence, we use information not only from the local wastewater viral concentration but also from the national prevalence estimates.

The two sets of LTLA-level random effects $U_i$ and $M_i$ are modelled as $U_i\sim \text{ Normal }(0,\sigma ^2_U)$ and $M_i\sim \text{ Normal }(0,\sigma ^2_M)$ respectively. We also investigated the use of an autoregressive structure on $U_i$ that allows local disease prevalence to vary smoothly over space^26^. However, there was no discernible difference in the nowcast quality between the two specifications. For $V_{it}$, we used $V_{it}\sim \text{ Normal }(0,\sigma ^2_V)$. A weakly informative prior $\text{ Uniform }(0,10)$ distribution was independently assigned to each random effect standard deviation, namely, $\sigma _U$, $\sigma _V$ and $\sigma _M$.

#### Implementation

Statistical inference is carried out in Stan^27^ via Markov chain Monte Carlo (MCMC) sampling. The two sub-models are jointly fitted, which allows the incorporation of different sources of uncertainty and the propagation of such uncertainty to the nowcast prevalence. A key source of uncertainty to capture is that associated with the de-biased prevalence and the wastewater viral concentration, both being outputs of their respective models with associated predictive distributions. The Sections on the national- and LTLA-level sub-models discuss the incorporation of the uncertainty associated with the de-biased prevalence through the likelihood. To account for the uncertainty associated with the wastewater estimates, we used multiple imputation^28^, whereby we performed the joint fitting of the two sub-models 50 times. For each model fit, we used a set of wastewater values, ${\tilde{w}}_{it}$ and ${\tilde{W}}_t$, randomly sampled from the joint posterior distribution of the wastewater model, hence retaining the inferred spatial and temporal correlation structures. We then pooled all the MCMC iterations from the 50 fits together to form the final predictive distributions for the nowcast prevalence. Model fitting with 50 fits took approximately 6 hours to complete using a single core on a MacBook Pro with M2 Pro chip. The computational time can be greatly reduced if the 50 fits are run on multiple cores. To investigate if 50 fits are sufficient, we also fitted the simplified data integration model 200 times with 200 sets of wastewater values. The parameter estimates, in terms of the posterior mean and the lower and upper bounds of the 95% credible interval, from the 50 fits are very close to those from the 200 fits (Figure 4 in the Supplementary Material). Thus, 50 fits are sufficient to encapsulate the uncertainty associated with the wastewater estimates. Figure 1 in Supplementary shows the directed acyclic graph of the full model structure.

### Setting for cross-validation and a set of comparative measures used

To evaluate the nowcast performance, we specified the first 20 weeks as the training period, with inputs provided by the weekly estimates of the wastewater viral concentration and de-biased COVID prevalence from all 307 LTLAs as well as their national-level estimates. Note that the LTLA-level prevalence data allow the estimation of two key terms in the LTLA-level sub-model: the between-LTLA variation in prevalence $U_i$ and the between-LTLA variation in the wastewater-prevalence relationship $M_i$ (see Eq. 2). The length of the training period was set to 20 weeks to ensure $U_i$ and $M_i$ could be estimated reliably. Specifically, we compared the estimates of $U_i$ and $M_i$ using 5, 10, 15, 20, 25 and 30 weeks of local prevalence data and found that 20 weeks gave a sufficient amount of data to yield estimates that were similar to those obtained using the entire 40 weeks of local prevalence data. The remaining 20 weeks of LTLA-weekly prevalence estimates were reserved for validation. We stress that the length of the training period is disease and geography specific, thus careful consideration is required when using this framework in a different setting. Hence, an evaluation of the stability of the estimates for $U_i$ and $M_i$ over a training period of varying lengths should be carried out.

In addition to the first 20 weeks of local data for training, national-weekly prevalence estimates were also required as model inputs for both the training period and the nowcast period. This ensures that the nowcast of local disease prevalence takes information from not only the spatially refined wastewater data but also the national-level prevalence estimates.

To compare the quality of the point estimates of the nowcast, we calculated the root mean square error, defined as

$$\begin{aligned} RMSE = \sqrt{\frac{1}{307\times 20}\sum _{i=1}^{307}\sum _{t=21}^{40}\left( p_{it}^{obs} - p_{it}^{nowcast}\right) ^2} \end{aligned}$$

where $p_{it}^{obs}$ and $p_{it}^{nowcast}$ are respectively the observed logit-transformed prevalence and the nowcast prevalence from a model. A lower RMSE indicates a more accurate set of nowcast prevalence.

To gauge how well a model estimates the nowcast uncertainty, we computed the coverage rate of the 95% credible interval and the average interval width:

$$\begin{aligned} coverage = \frac{1}{307\times 20}\sum _{i=1}^{307}\sum _{t=21}^{40} I \left( cp_{it}^{nowcast, lower} \le p_{it}^{obs} \le cp_{it}^{nowcast, upper}\right) \end{aligned}$$

$$\begin{aligned} width = \frac{1}{307\times 20}\sum _{i=1}^{307}\sum _{t=21}^{40} \left( cp_{it}^{nowcast, upper} - cp_{it}^{nowcast, lower}\right) \end{aligned}$$

where $cp_{it}^{nowcast, lower}$ and $cp_{it}^{nowcast, upper}$ are the lower and the upper bounds of the 95% credible interval for the nowcast prevalence $p_{it}^{nowcast}$ and $I(\cdot )$ is the indicator function that returns 1 if the argument is true and 0 otherwise. A model with a coverage rate close to the nominal level of 95% provides reliable estimates of the predictive uncertainty. A model with a lower average interval width gives nowcast that are more certain. Taken together, we employed these three measures, RMSE, coverage rate and interval width, to examine the quality of the probabilistic nowcasts across different models.

#### Different nowcast models

The key features of the different nowcast models considered in this study are summarised in Supplementary Table 1. The specification of each model is discussed in Section A in the Supplementary.

### Ethics

The Alan Turing Institute Ethics Advisory Group provided guidelines for this study’s procedures and advised that Health Research Authority approval is not required for this research.

### Supplementary Information

Supplementary Information.

## Data Availability

The LTLA-weekly level wastewater viral concentration estimates and the de-biased COVID-19 prevalence are available on the GitHub repository https://github.com/gqlNU/wwprev. Also available on the repository are the R scripts for model fitting and data/result visualisation.
