# Supplementary material for: Integrating wastewater and randomised prevalence survey data for national COVID surveillance
Source: Sci Rep. 2024 Mar 1;14:5124. doi: 10.1038/s41598-024-55752-9 (PMC10907376; doi:10.1038/s41598-024-55752-9)
Supplement: Supplementary file 1 — Supplementary Information. [file 41598_2024_55752_MOESM1_ESM.pdf]

Integrating wastewater and randomised prevalence survey data for national COVID surveillance

Guangquan Li, Peter Diggle and Marta Blangiardo

Supplementary Material

Contents

A Different nowcast models **1**

B The directed acyclic graph for the data integration model **2**

C Comparing the interval widths between the full model and the simplified model **4**

D Parameters of interest in the simplified model **5**

E Comparison of parameter estimates from fitting the data integration model 50 times vs 200 times **7**

A Different nowcast models

Table 1 summarises the key specifications of the different models for nowcasting local prevalence considered in the paper. Models 1-3 present the different versions of the data integration framework where Model 1 allows the wastewater-prevalence relationship to vary over both space and time while Model 2, a simplified version of Model 1, assumes the relation to vary only spatially but not temporally. Model 3 extends Model 2 by incorporating two LTLA-level covariates, the English Index of Multiple Deprivation (IMD) and the percentage of Black, Asian, and minority ethnic (BAME) people in the population. Model 4 excludes the use of the wastewater viral concentration estimates and thus its nowcast of local prevalence is a function of the national estimates $\alpha + B_t$ plus the estimated local variation

U_i. Model 5 produces local prevalence nowcast using the wastewater viral concentrations only, with no anchoring from the national level prevalence.

In the Results Section in the main paper, we considered two scenarios where the spatially coarse prevalence estimates were available at the regional level. For those scenarios, we fitted Model 2 to each of the 9 English regions separately.

B The directed acyclic graph for the data integration model

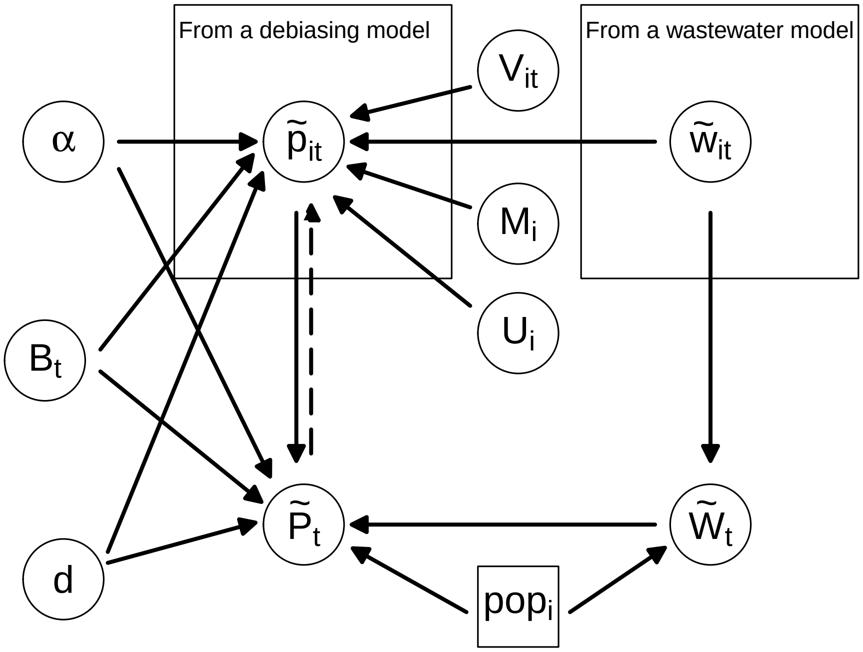

Figure 1: The directed acyclic graph for the data integration model with spatially varying relation between wastewater viral concentration and prevalence, i.e. the simplified model specified in the Methods Section in the main paper. The dashed arrow going from $\tilde{P}_t$ to $\tilde{p}_{it}$ emphasises the contribution of the national prevalence estimates when nowcasting local prevalence.

Table 1: Key specifications of the different models for nowcasting local disease prevalence

Model	The LTLA-level sub-model with the likelihood $p_{it} \sim Normal(\mu_{it}, \sigma_{it}^2)$	The national-level sub-model with the likelihood $P_t \sim \text{Normal}(\mu_t, \sigma_t^2)$
1. The full model	$\mu_{it} = (\alpha+U_i)+(B_t+V_{it})+(d+D_t+M_i)\cdot\tilde{w}_{it}$	$\mu_t = \alpha + B_t + (d + D_t) \cdot \tilde{W}_t$
2. The simplified model	$\mu_{it} = (\alpha+U_i)+(B_t+V_{it})+(d+M_i)\cdot\tilde{w}_{it}$	$\mu_t = \alpha + B_t + d \cdot \tilde{W}_t$
3. The simplified model + covariates (IMD and BAME)[a]	$\mu_{it} = (\alpha+U_i+\beta_1 \cdot \text{IMD}_i + \beta_2 \cdot \text{BAME}_i) + (B_t+V_{it})+(d+M_i)\cdot\tilde{w}_{it}$	$\mu_t = \alpha + B_t + d \cdot \tilde{W}_t$
4. Without wastewater	$\mu_{it} = (\alpha+U_i)+(B_t+V_{it})$	$\mu_t = \alpha + B_t$
5. Without national prevalence	$\mu_{it} = (\alpha+U_i)+(B_t+V_{it})+(d+M_i)\cdot\tilde{w}_{it}$	No national sub-model in this version

[a] A weakly informative prior $\text{Normal}(0, 10^6)$ was independently assigned to β_1 and β_2.

C Comparing the interval widths between the full model and the simplified model

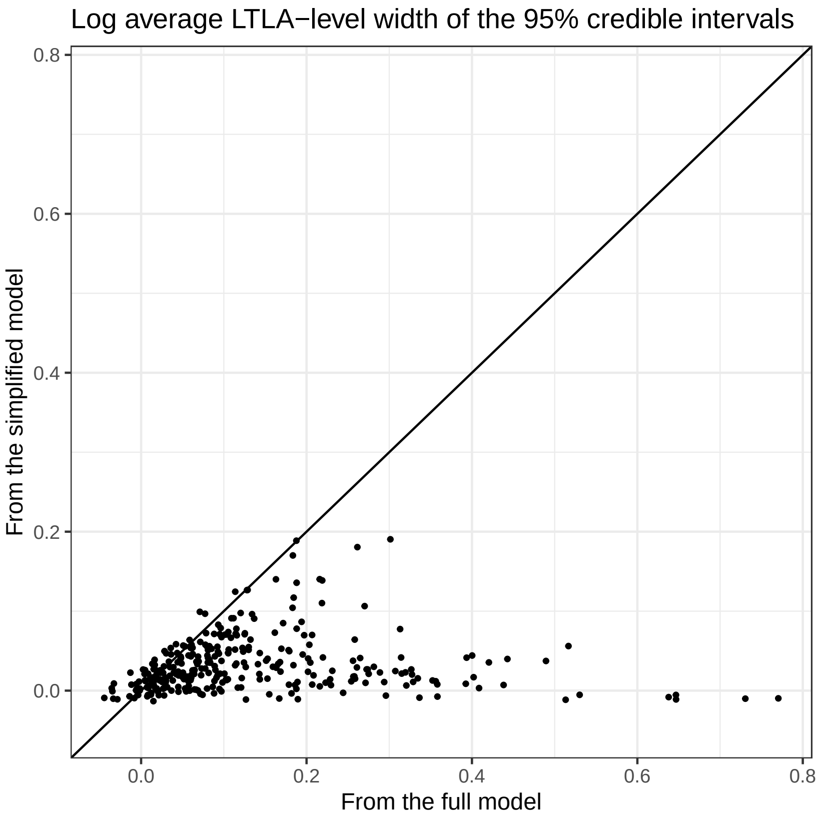

Figure 2: Comparing the log-transformed width of the 95% credible intervals from the simplified model against those from the full model. The interval width of each LTLA is averaged over the 20 week of validation period. There are 261 (out of 307) LTLAs where the average interval lengths from the simplified model are shorter than those from the full model. For 46 LTLAs, the interval lengths from the simplified model are longer but the differences in interval width between the two models are almost negligible.

D Parameters of interest in the simplified model

Parameter	Description	Posterior estimate
α	Intercept for the LTLA-weekly logit-transformed prevalence	-4.39 (-4.44, -4.36)
B_t	Temporal random effects describing the national temporal pattern of the prevalence	See Figure 3(A)
U_i	LTLA-level random effects capturing the between-LTLA variation in the logit-transformed prevalence	See Figure 3(B)
d	The overall relationship between prevalence and wastewater viral concentration at the national level	0.22 (0.18, 0.26)
M_i	LTLA-level random effects capturing the between-LTLA variation in the prevalence-wastewater relationship	See Figure 3(C)
σ_B	Standard deviation of the temporal random effects B_t	0.22 (0.18, 0.29)
σ_U	Standard deviation of the LTLA-level random effects U_i	0.23 (0.20, 0.26)
σ_M	Standard deviation of the LTLA-level random effects M_i	0.10 (0.09, 0.11)
σ_V	Standard deviation of the LTLA-weekly random effects V_{it}	0.23 (0.22, 0.24)

Table 2: Key parameters in the simplified data integration model. The posterior mean and 95% credible interval are reported for some parameters. Figure 3 visualises the posterior estimates of some random effect components.

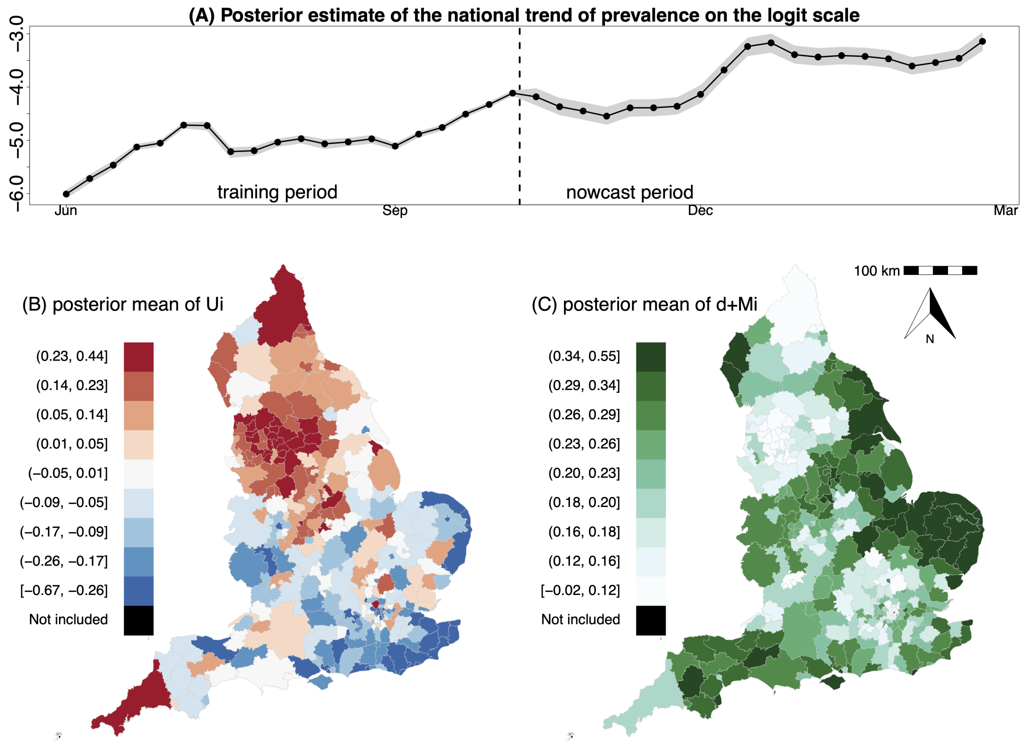

Figure 3: Visualisation of parameter estimates from the simplified data integration model under the setting where the first 20 weeks of LTLA-weekly prevalence and wastewater data are used to train the model while the second 20 weeks is the nowcast period in which the trained model uses the LTLA-weekly wastewater data and the weekly national prevalence estimates to produce weekly prevalence at the LTLA level. Panel (A): the estimated national trend, the posterior means with the 95% uncertainty band, for the logit-transformed prevalence, $\alpha + B_t$ with $t = 1, ..., 40$ weeks; (B) a map of the posterior mean of U_i, the LTLA-level random effects capturing the between-LTLA variation in prevalence on the logit scale; and (C) a map of the posterior mean of $d + M_i$, showing the spatial variation in the prevalence-wastewater relationship - a darker green colour suggests a stronger prevalence-wastewater association. The digital vector boundaries for Local Authority Districts in England were obtained from the Office for National Statistics Open Geography Portal (Source: Office for National Statistics licensed under the Open Government Licence v.3.0 and contains OS data © Crown copyright and database right 2022). The map in this figure was produced in R (version 4.3.1; https://www.R-project.org/). The R script to produce this figure can be found on the GitHub repository https://github.com/gqlNU/wwprev. 6

E Comparison of parameter estimates from fitting the data integration model 50 times vs 200 times

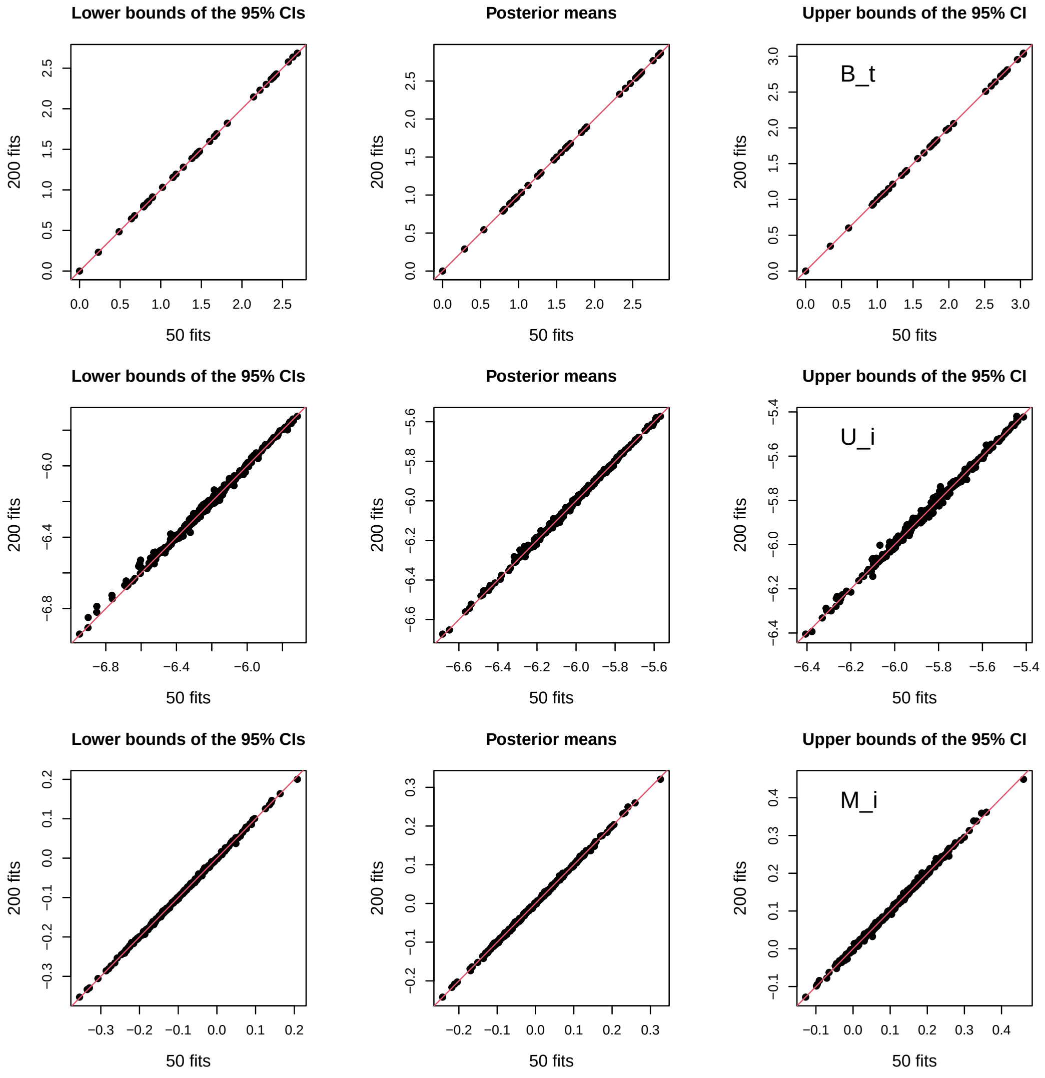

Figure 4: We fitted the data integration model to 200 sets of wastewater values generated from the joint posterior distribution of the wastewater model and compared the parameter estimates against those obtained by fitting the same model to 50 sets. Three key sets of parameteres are compared: B_t (the top row; $t = 1, ..., 40$)), the pattern of the national prevalence trend, U_i (the middle row; $i = 1, ..., 307$), the between-LTLA prevalence variation, and M_i (the third row), the between-LTLA variation in the prevalence-wastewater relationship. For each set of random effects, we compare the lower and upper bounds of the 95% credible intervals (CIs; the first and the third columns, respectively) and their posterior means (the second column). For ease of comparison, a diagonal line in red is shown in each figure. The estimates are very similar between the 50 and 200 fits, confirming that 50 fits of the model are sufficient to incorporate and propagate the uncertainty in the wastewater estimates.
